# Post‐Acupuncture Acute Cervical Spinal Epidural Hematoma With Hemiplegia Misdiagnosed as Cerebral Infarction: A Case Report and Literature Review

**DOI:** 10.1002/ccr3.70779

**Published:** 2025-08-14

**Authors:** Pan Wang, Ping Luo, Zhong Xiang, ZhiWu Chen, Meng Zhang, Fan Zhou, Bin Hu

**Affiliations:** ^1^ Department of Orthopaedics The Fourth Hospital of Changsha, Changsha Hospital of Hunan Normal University Changsha China

**Keywords:** acute cervical spinal epidural hematoma, cerebral infarction, misdiagnosed, post‐acupuncture

## Abstract

This case report describes a rare but serious complication termed post‐acupuncture acute cervical spinal epidural hematoma (paACSEH). The patient presented with right‐sided hemiplegia and cervical pain following acupuncture therapy. Initial misdiagnosis as cerebral infarction led to inappropriate alteplase thrombolytic therapy, which was promptly discontinued after CT imaging confirmed cervical spinal epidural hematoma. Given the patient's concurrent clopidogrel therapy and recent thrombolysis, surgical intervention was considered high‐risk for rebleeding. Conservative management was subsequently adopted due to neurological stabilization, resulting in favorable clinical outcomes. This case underscores the critical importance of considering paACSEH in patients with: (1) recent cervical acupuncture history; and (2) acute neurological deficits (particularly hemiplegia with cervical pain in the absence of speech or consciousness impairment). Our findings suggest that conservative treatment may yield satisfactory recovery, potentially with faster functional restoration than surgical intervention, in cases demonstrating early neurological improvement or having surgical contraindications.

**Summary**

- Post‐acupuncture cervical epidural hematoma mimics stroke but lacks speech/consciousness impairment.
- Urgent spinal imaging prevents misdiagnosis and harmful thrombolysis.
- Conservative management is viable if neurological deficits stabilize early.

## 1 Introduction

paACSEH also known as post‐acupuncture traumatic cervical subdural hematoma, is an extremely rare clinical condition. It typically presents with rapidly progressive symptoms of spinal cord compression, leading to paraplegia within a short period. Due to its low incidence, paACSEH is prone to being missed, misdiagnosed, or delayed in treatment. If not promptly identified and managed, it can result in irreversible neurological damage in patients. In recent years, with the widespread use of anticoagulants and acupuncture techniques, reports of paACSEH have gradually increased. The primary treatment for paACSEH is surgical intervention, while recovery through conservative management is relatively uncommon.

## 2 Case History/Examination

The patient was a 73‐year‐old female admitted to the hospital due to weakness and numbness in the right limb for over 1 h. According to the patient, she had undergone acupuncture treatment on the neck and shoulder at a community hospital for dizziness 1 h prior. Half an hour after the acupuncture session, she noticed weakness and numbness in her right limb and was urgently sent to the hospital. The patient had a history of hypertension and coronary heart disease and had been on long‐term clopidogrel anticoagulation therapy.

## 3 Methods (Differential Diagnosis, Investigations, and Treatment)

**FIGURE 1:**
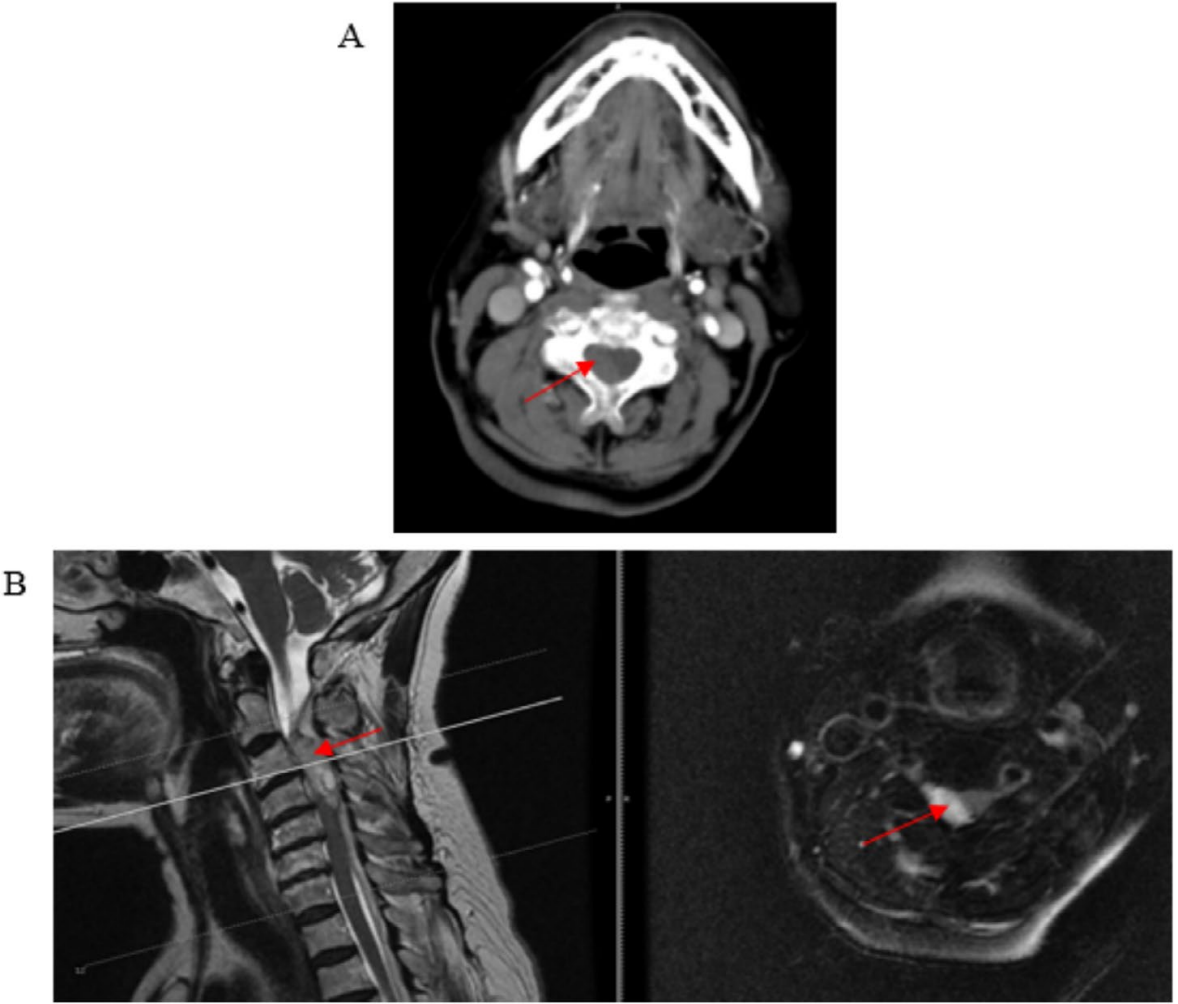
(A) Cervical CT scan reveals an epidural hematoma with significant compression and flattening of the spinal cord. (B) Cervical MRI demonstrates an epidural hematoma within the cervical spinal canal, showing leftward displacement of the spinal cord at the corresponding levels, most notably at C2–5.

On admission, physical examination revealed muscle strength of the right upper limb was 0/5, right lower limb was 1/5, and left limbs were 5/5. Muscle tone was normal in all limbs. The right limb exhibited decreased sensation with numbness, hyperreflexia, and positive Babinski and Hoffman signs. No significant abnormalities were observed in the left limb. The patient was suspected of having an acute cerebral infarction and was treated with alteplase thrombolysis by the neurology department. During thrombolysis, a CT scan revealed a C2‐5 spinal epidural hematoma (Figure 1A). Thrombolysis was immediately discontinued, and a consultation with our department was requested. After evaluation, an emergency cervical MRI was performed (Figure 1B), which confirmed a C2‐5 spinal epidural hematoma with severe spinal cord compression. Two hours after transfer to our department, a physical examination showed muscle strength of the right upper limb was 1/5 and the right lower limb was 3/5. Considering the patient's history of clopidogrel anticoagulation and alteplase thrombolysis, the risk of surgical re‐bleeding was high. Additionally, the patient's muscle strength had significantly improved compared to 2 h prior, suggesting that active bleeding had stabilized. Therefore, after comprehensive consideration, conservative treatment was initiated, including mannitol for dehydration and methylprednisolone pulse therapy to prevent spinal cord edema.

## 4 Conclusion and Results

**FIGURE 2:**
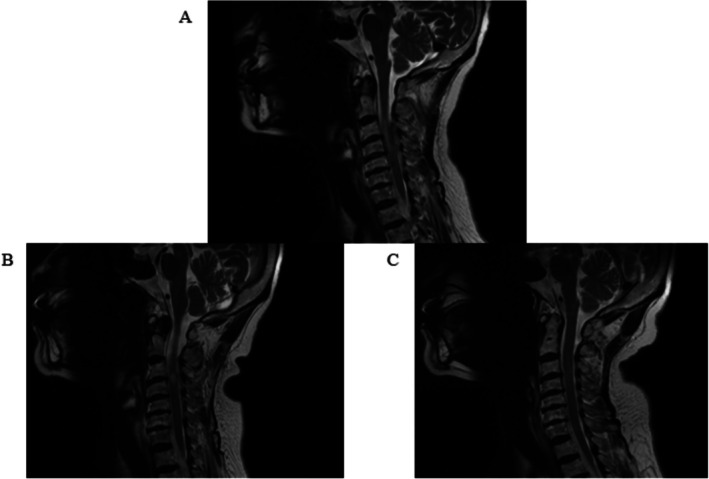
(A) The epidural hematoma shows significant absorption compared to previous imaging. The leftward displacement of the spinal cord at the C2‐5 levels has improved, with evidence of spinal cord degeneration at the C3‐4 level. (B) Further absorption of the epidural hematoma is observed, with continued improvement in spinal cord compression at the corresponding levels. The area of spinal cord degeneration at the C3‐4 level has reduced compared to prior imaging. (C) The epidural hematoma has completely resolved, and no evidence of spinal cord degeneration at the C3‐4 level is visible.

On the second day, physical examination revealed muscle strength of the right upper limb was 3/5 and the right lower limb was 4/5. A repeat cervical MRI showed significant absorption of the hematoma, accompanied by C3‐4 spinal cord degeneration and spinal cord injury (Figure 2A). One week later, physical examination showed muscle strength of the right upper limb was 4/5 and the right lower limb was 5/5. Numbness and sensory deficits in the right limb had largely resolved. A repeat cervical MRI demonstrated further absorption of the epidural hematoma and improved spinal cord compression at the corresponding levels. The patient was able to walk and requested discharge (Figure 2B). At the 20‐day follow‐up, physical examination revealed normal muscle strength in the right limb. A cervical MRI showed complete absorption of the epidural hematoma, and the previous abnormal signal in the cervical spinal cord was no longer visible (Figure 2C).

## 5 Discussion

Spinal epidural hematoma (SEH) was first reported by Jackson in 1869 and remains a rare clinical condition [1]. The typical clinical presentation includes sudden onset of sharp neck pain accompanied by rapidly progressive symptoms of spinal cord compression, often leading to paraplegia within a short period [2]. Due to its overlapping clinical features with acute cerebral infarction [3, 4], coupled with insufficient awareness of the condition, SEH is frequently misdiagnosed or delayed in treatment, resulting in irreversible neurological deficits [5]. Previous literature indicates that most SEH cases are associated with identifiable risk factors, including spinal vascular malformations (such as aneurysms, dural arteriovenous fistulas, and toxemia of pregnancy), iatrogenic spinal interventions (e.g., acupuncture, spinal manipulation, and epidural block), coagulation disorders, and the use of anticoagulant medications [6, 7, 8, 9]. In recent years, with the widespread application of anticoagulant medications, the incidence of SEH has shown an increasing trend. Notably, Guler A et al. [10] reported a representative case in which a patient receiving dual antiplatelet therapy developed cervical epidural hematoma following persistent cough, ultimately requiring surgical intervention.

For most individuals, acupuncture is considered a safe procedure. The majority of acupuncture‐related complications are harmless, transient, and mild, including minor bleeding or hematoma at the needle insertion site and pain during needle insertion or removal [11, 12]. Severe complications directly caused by acupuncture are rare [13] and include infections, pneumothorax, neurological injuries (central and peripheral nervous systems), cardiac injuries (cardiac tamponade), and vascular injuries [14]. PaACSEH, defined as traumatic cervical spinal epidural hematoma following acupuncture, is an extremely rare clinical entity. However, with the increasing use of anticoagulants and acupuncture techniques, reports of paACSEH have gradually risen in recent years.

We conducted a computerized search of the Embase, Web of Science, PubMed, and Cochrane Library databases using the keywords “acupuncture complications” and “cervical spinal epidural hematoma.” In addition to the case reported here, we identified six other well‐documented cases [15, 16, 17, 18, 19] (Table 1).

**TABLE 1:** Patient's Demographics.

	Case 1 [16]	Case 2 [18]	Case 3 [15]	Case 4 [15]	Case 5 [17]	Case 6 [14]	Present case
Sex/Age	F 58 y	M 69 y	F 54 y	F 38 y	M 38 y	M 64 y	F 73 y
Diseases before acupuncture	Cervical spondylosis	Musculoskeletal pain	Neck pain	Headache and shoulder pain	Stiff neck	Sciatica	Dizziness
Time*	6 h	4 h	A few hours	—	10 min	8 h	30 min
Pain**	Yes	No	—	—	—	Yes	Yes
Sensory changes**	Yes	No	Yes	—	Yes	Yes	Yes
Grade muscolar** power	R Grade I; L Grade IV	R Grade III; L Grade V	R ‐; L Grade III	R ‐； L Decreased	R Grade V; L Grade 0	R Grade V; L Grade II	R Grade 0; L Grade V
Anal sphincter tone**	Decreased 50%	Normal	—	—	Decreased	Normal	Normal
Level of involvement	C3‐T1	C3‐C5	C3‐7	C5‐T2	C2‐4	C2‐T12	C3‐C5
Treatment	Laminectomy	Laminectomy	Laminectomy	Laminectomy	Laminectomy	Conservative	Conservative
Result	Complete recovery	Complete recovery	Recovery Grade IV	Recovery Grade IV	Recovery Grade IV	Complete recovery	Complete recovery

*Note:* The grading muscle power of the cases was evaluated with the Medical Research Council scale from 0 to 5.

* Time between acupuncture and onset of symptoms.

** At the moment of admission to the hospital.

The mean age of the patients was 57 years (range: 38–73 years), with three males and four females. In all cases, acupuncture was performed to relieve neck pain or stiffness. The time interval between acupuncture and symptom onset ranged from 10 min to several hours. Two patients exhibited anal sphincter relaxation [17, 18], two patients [15, 17] and the present case reported significant neck pain, and four patients [15, 16, 17, 18], along with the present case, experienced unilateral sensory deficits. All cases presented with the typical symptom of hemiplegia on one side. Except for one case where the hematoma extended from C2 to T12 [15], the hematoma in the remaining cases was confined to 2, 4, or 6 spinal segments. With the exception of one case that underwent conservative treatment due to extensive hematoma and rapid improvement of paraplegia symptoms [15], all other cases were treated with surgical laminectomy and decompression. In all cases, significant recovery of motor function was observed following either conservative or surgical treatment.

In previously reported cases, paACSEH has been regarded as a neurosurgical emergency requiring immediate intervention to prevent irreversible neurological damage [18]. Early recognition and surgical decompression have been shown to significantly improve neurological outcomes [20]. In the present case, the patient presented with severe hemiplegia, and surgical decompression was initially considered to relieve spinal cord compression. However, due to the patient's history of clopidogrel use and thrombolysis with alteplase for a misdiagnosis of acute cerebral infarction, surgical intervention carried a high risk of intraoperative bleeding and postoperative hematoma recurrence. Fortunately, during the period when the patient and their family were considering the surgical risks, the patient's neurological symptoms showed significant improvement, providing an opportunity for conservative management. After thorough discussion with the patient's family, conservative treatment was initiated with readiness for surgical intervention if neurological deterioration occurred. By the second day, the patient's motor function had further improved, and a follow‐up cervical MRI revealed significant absorption of the C3‐5 epidural hematoma, accompanied by C3‐4 spinal cord degeneration and injury. One week later, the patient's motor function had nearly fully recovered, and they were able to walk. A repeat cervical MRI showed further absorption of the epidural hematoma and reduced spinal cord compression. At the 20‐day follow‐up, the patient's right‐sided muscle strength had returned to normal, and the cervical MRI demonstrated complete resolution of the epidural hematoma and the previously observed abnormal spinal cord signal. A review of the literature revealed that Domenicucci et al. [15] reported a case of paACSEH with a massive C2‐T12 epidural hematoma that nearly completely resolved by the fourth day with conservative treatment. The patient was discharged on the sixth day and eventually achieved full recovery. In contrast, the fastest recovery time for surgically treated patients was 2 weeks [18], suggesting that conservative management may lead to faster recovery in selected cases of paACSEH. Therefore, we recommend that conservative treatment be considered for patients with early neurological improvement within 24 h or those with contraindications to surgery.

Additionally, it is noteworthy that the present case was initially misdiagnosed as acute cerebral infarction and treated with alteplase thrombolysis. Fortunately, the epidural hematoma did not rebleed, and the patient's neurological symptoms did not worsen. Hemiparesis is a common presentation of SEH and is often misdiagnosed as acute cerebral infarction [21]. Park et al. [19] reported a case of paACSEH initially suspected to be acute cerebral infarction and treated with antiplatelet agents and aspirin. Despite timely surgical decompression 36 h later, the patient required 3 months to achieve full motor recovery. These findings highlight that, in most misdiagnoses, neurologists tend to focus on hemiparesis while overlooking key diagnostic clues such as a history of acupuncture, neck pain, and the absence of speech or consciousness impairment, which can help differentiate paACSEH from acute cerebral infarction. Therefore, we recommend that paACSEH be considered in patients with hemiparesis who have a history of acupuncture, neck pain, and no speech or consciousness impairment. Cervical CT and MRI should be promptly performed to confirm the diagnosis and avoid delays in treatment.

## Author Contributions

**Pan Wang:** investigation, methodology. **Ping Luo:** writing – original draft. **Zhong Xiang:** conceptualization. **ZhiWu Chen:** data curation. **Meng Zhang:** data curation. **Fan Zhou:** data curation. **Bin Hu:** writing – original draft, writing – review and editing.

## Ethics Statement

The authors have nothing to report.

## Consent

Written consent has been taken from the patient for the publication of this case report.

## Conflicts of Interest

There are no conflicts of interest associated with the submission of this manuscript, and all the authors approved the manuscript for publication. On behalf of my co‐authors, I would like to state that the work described is original research that has not been previously published and was not considered for publication in whole or in part elsewhere. All authors listed have approved the accompanying manuscript

## Data Availability

Data available on request due to privacy/ethical restrictions: The data that support the findings of this study are available on request from the corresponding author. The data are not publicly available due to privacy or ethical restrictions.
